# Precision radiotherapy for non-small cell lung cancer

**DOI:** 10.1186/s12929-020-00676-5

**Published:** 2020-07-22

**Authors:** Wen-Chi Yang, Feng-Ming Hsu, Pan-Chyr Yang

**Affiliations:** 1 Division of Radiation Oncology, Department of Oncology, National Taiwan University Hospital, No. 7, Chung-Shan South Rd, Taipei, Taiwan; 2 Graduate Institute of Oncology, National Taiwan University College of Medicine, Taipei, Taiwan; 3 Department of Internal Medicine, National Taiwan University Hospital, No.1 Sec 1, Jen-Ai Rd, Taipei, 100 Taiwan

**Keywords:** Precision radiotherapy, Non-small cell lung cancer, Dosiomics, Panomics, Radiomics

## Abstract

Precision medicine is becoming the standard of care in anti-cancer treatment. The personalized precision management of cancer patients highly relies on the improvement of new technology in next generation sequencing and high-throughput big data processing for biological and radiographic information.

Systemic precision cancer therapy has been developed for years. However, the role of precision medicine in radiotherapy has not yet been fully implemented. Emerging evidence has shown that precision radiotherapy for cancer patients is possible with recent advances in new radiotherapy technologies, panomics, radiomics and dosiomics.

This review focused on the role of precision radiotherapy in non-small cell lung cancer and demonstrated the current landscape.

## Introduction

Precision medicine is an emerging new era of future healthcare [1]. It has become feasible because of advances in next generation sequencing (NGS) and panomics technologies as well as the integration of large-scale biologic databases and artificial intelligence to identify biomarkers, stratify patients and precisely guide clinical practices. It has significantly improved the treatment outcome of human diseases especially in cancer therapy.

Non-small cell lung cancer (NSCLC) is one of the examples of precision medicine being most successfully applied [2]. The standard guidelines for precision management in NSCLC recommend stratifying patients by histology (adenocarcinoma versus squamous cell carcinoma or large cell carcinoma), followed by gene testing of druggable driver mutations (EGFR, ALK, ROS1, BRAF, NTRK, etc.) for target therapy. If patients were negative for druggable mutations, PDL-1 immunohistochemistry and/or tumor mutation burden will be tested to assess the suitability of immune checkpoint inhibitor therapy. The implementation of precision therapy has significantly improved the treatment outcome of NSCLC [3].

Radiotherapy is an effective anti-cancer treatment for nearly half of all cancer patients [4]. In NSCLC, radiotherapy may serve as a definitive treatment to early stage inoperable tumor [5] and local advanced disease [6, 7]. With the improvement in the survival of NSCLC cancer patients, the use of radiotherapy has become more common. Radiotherapy was also the greatest increase in Medicare expenditures between 2003 and 2009 in the U.S. [8]. It was used not only in definitive treatment but also in palliative therapy. For example, better local control can be achieved with thoracic re-irradiation for NSCLC [9]. Stereotactic radiosurgery (SRS) has been becoming the standard management to treat NSCLC patients with limited brain metastases [10]. The idea of customized systemic precision management of NSCLC has been extended to combine local radiotherapy with the current standard options of chemotherapy, targeted therapy or immunotherapy. In the radical-palliative setting, stereotactic ablative radiotherapy (SABR) to oligometastatic NSCLC lesions was proved to be effective with promising outcome. Iyengar P. et al. conducted a phase two trial to compare maintenance chemotherapy alone versus SABR followed by maintenance chemotherapy for patients with limited (up to 5 metastatic sites) metastatic NSCLC. The results showed SABR arm tripled the progression-free survival [11, 12].

**Fig. 1:**
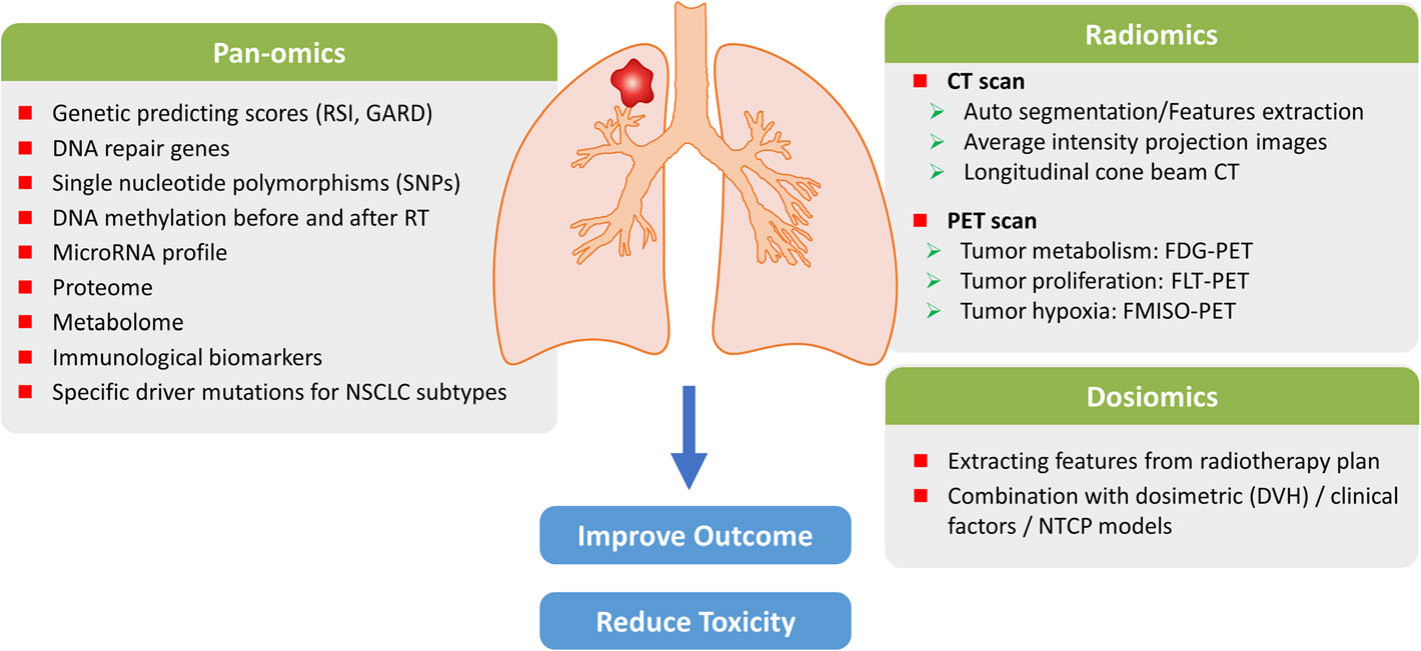
Landscape of Precision Radiotherapy in NSCLC

The term precision radiotherapy aims to stratify and precisely treat each individual cancer patient, using state-of-the-art new radiotherapy technology and biomarkers to improve treatment outcome and reduce adverse effects. The advances of radiotherapy technology make precision radiotherapy applicable. Technological advances provided better treatment modalities, although did not necessarily individualize the treatment plan for each patient. Emerging investigations and reviews have raised the attention of true personalized radiotherapy [13, 14] for different cancer types. In this article, we summarize the current landscape of precision radiotherapy for NSCLC (Fig. 1), focusing on recent advances in radiotherapy technology, radiomics and dosiomics, as well as biomarkers derived from panomics that may improve treatment outcome and reduce potential adverse effects **(**Table 1**)**.

**Table 1:** Biomarkers predicting outcome and toxicity in radiotherapy for NSCLC

Types of Biomarkers	Outcome predictors	Toxicity predictors
Panomics		
Genomics	Genomic predicting scores: Radiosensitiviy Index (RSI), Genomic-adjusted radiation dose (GARD) Lung adenocarcinoma associated gene: KRAS Squamous cell carcinoma associated genes: KEAP1, NFE2L2	DAN repair genes: ATM Other mutated genes: PTEN, RB1, TP53 …
SNPs	DNA repair gene sites: XRCC1, BRCA1, and ERCC1	DNA repair gene sites: ATM, RAD51, XRCC family, LIG4, MTHFR Inflammatory gene sites: TGFβ1 Immune modulated gene sites: CBLB
Epigenetics	DNA methylation profile, IGFBP-3 Micro RNA: p53 regulation, RAD51 regulation (MiR-34a)	–
Proteome /Metabolome	–	Serum inflammatory biomarkers: TGFβ, IL-1, KL-6, IL-6 IL-8, PDGF, TGFα, TNFα, CXCL10 (IP-10), CCL2 (MCP-1), Eotaxin, and TIMP-1 Novel proteins identified by mass spectroscopy:C4BPA, VTN, α2M, CO4A, CO5
Immunological markers	Neutrophil-to-lymphocyte ratio (NLR), platelet-to-lymphocyte ratio (PLR), neutrophil count, lymphocyte count	Neutrophil-to-lymphocyte ratio (NLR)
Radiomics		
CT scan	Post SABR local recurrence prediction: enlarging opacity at 12 months after SABR, bulging margin, loss of linear margin and air bronchogram lossPost SABR recurrence free survival: tumor size, pleural retraction, vessel attachment For adaptive RT during chemoradiation: LARTIA trial	Combination of radiomic signatures predict radiation pneumonitis
PET	FDG-PET: Max SUV, metabolic volume, total lesion glycolysis FLT-PET: sensitive than FDG-PET to predict tumor response F-MISO-PET: identify radioresistant area for adaptive treatment	FLT-PET: predict hematological toxicity (bone marrow)
Dosiomics		Dosiomic information + dosimetric information (DVH) + clinical factors better predict radiation pneumonitis

Abbreviations: *NSCLC* Non-small cell lung cancer. *CT* Computed tomography. *PET* Positron emission tomography. *SABR* Stereotactic ablative radiotherapy. *DVH* Dose volume histogram

### Technological advancements for precision radiotherapy

Precision radiotherapy was used for decades to describe the improvement of technology in medical ionizing irradiation. The development of radiotherapy techniques can be characterized into several aspects. First, the radiation machine improved from a low-voltage X-ray generator to a high voltage X-ray linear accelerator. Second, in addition to conventional fractionated treatment, the idea of high-dose-single-shot stereotactic radiosurgery has also been developed to achieve better tumor control [5]. Through the development of planning systems and hardware, such as multi-leaf collimators, intensity modulated radiotherapy (IMRT) can be administered with adaption to the tumor location and surrounding normal tissues, which may improve tumor control and reduce toxicity [15]. For particle treatment, commercialized cyclotrons and synchrotrons make proton treatment more popular in real world practice. The development of Monte Carlo dose simulation has helped to overcome the treatment uncertainty in proton therapy [16]. Heavy particles are also under investigation and may be beneficial in some challenging cases [17]. Another improvement in radiotherapy is image registration modality for delivery precision. In-room cone beam CT on a linear accelerator improved the treatment accuracy. Some immobilization devices associated with adaptive software for breath control and gating system, also improve the treatment accuracy for moving targets, especially lung tumors. A recent recommendation was proposed by the European Organization for Research and Treatment of Cancer (EORTC) group, which provided a suggestion to provide high precision radiotherapy especially for lung cancer patients [18].

#### Radiomics

Radiomics is an emerging field in cancer treatment. For NSCLC, chest images are necessary for diagnosis and follow up, which makes NSCLC a good candidate for radiomics investigations [19]. In general, quantitative data from images for tumors were mined automatically to correlate with tumor behavior, treatment response, and clinical prognosis. The predictive power of radiomics provides a great opportunity for a non-invasive approach for precision radiotherapy. Without invasive procedures, tissue, blood or fluid samples, tumor characteristics can be represented from images themselves. Improvements in data processing tools and deep learning techniques helpinterpret data more efficiently and make clinical use possible. The major imaging methods used in NSCLC radiomics are CT and PET scans. The ideas between these two images are different. The tumor structure, shape, and compaction on a CT scan can be associated with tumor biological behavior and clinical outcome. In contrast, PET scan is a functional imaging tool, and the use of different tracers can directly show distinct biological features of disease.

#### Computed tomography (CT)

CT is the most common tool in radiomics studies for lung cancer. In the radiotherapy field, the main utilization of CT radiomics includes auto-segmentation and feature extractions [20–22]. For radiotherapy treatment of lung cancer, one of the most challenging issues is consistent contouring for an organ at risk (OAR) and accurate delineation for the gross tumor volume (GTV). The Thoracic Auto-Segmentation Challenge organized at the 2017 Annual Meeting of American Association of Physicists in Medicine (AAPM) reported the outcome of thoracic auto-segmentation with different approaches. In general, lung and heart cane be segmented consistently in most algorithms, while deep learning can better delineate the esophagus. This tool improves the treatment quality and precision [23]. As a moving target, the blurred border of lung tumors makes accuracy in contouring difficult. Besides, the collapsed lung field, pleural traction, and regional bronchus and vessels were not easy to differentiate from true GTV. A semi-automated method with three-dimensional lung CT was developed [24]. The GrowCut algorithm, an interactive region segmentation strategy, was reported to be able to reduce inter-physician differences in lung tumor contouring.

More popular issues are the feature extraction andprognostic or predictive values from different radiomic features. Features, which include lesion shape, intensity, texture and wavelet, together with location, can be extracted [21]. Longitudinal pattern changes were used to associate the response to radiotherapy. Definitive stereotactic ablative radiotherapy (SABR) is a treatment of choice for early stage lung cancer. For medically inoperable NSCLC patients with impaired lung function (for example, FEV1 < 40% predicted), SABR provided excellent outcme and became the standard of care [5]. Without other intervention procedures, SABR might be the least confounded treatment modality to investigate radiomics in radiotherapy treatment outcome. There are several applications of radiomics in NSCLC patients receivving SABR, and these include the assessment of early recurrence post SABR, the correlation between pretreatment radiomic features and clinical outcome, and the prediction of radiation induced lung toxicity.

A retrospective series analyzed the radiomic features associated with a higher risk of local and distant recurrence. CT density changes are common after SABR. In a systemic review, high risk features related to local recurrence were identified including enlarging opacity at different time points after SABR, bulging margin, loss of linear margin and air bronchogram loss [25]. These features were validated [26, 27] in other series. Enlargement of opacity 12 months after SABR was considered most predictive compared with other parameters. Opacity change in the primary lesion within 1 year may be related to post-irradiation change and subacute radiation pneumonitis. Considering that the lung is a moving organ, it was reported that average intensity projection images (AIP) are better than free breathing CT in a lung radiomics study [28] for predicting the treatment outcome. The main clinical implication is to help physicians makie decisions for the diagnosis of local recurrence. In a retrospective study, physician assessment and a radiomic prediction tool for local recurrence diagnosis were compared. A high false negative rate was noticed in the physician assessment group [29]. With radiomic prediction assistance, local recurrence after SABR can be identified earlier than physician’s assessment.

Radiomic features extracted from pre-treatment CT scans can be used to associate with clinical outcomes such as progression-free survival and overall survival [30–34]. Radiomic features from images were sorted into clusters and correlated with survival outcome. Li Q et al. [30] established a prognostic model incorporating clinical and genomic features. In addition to tumor size, an extracted feature from histogram analysis, measured the energy of Housfield unit (HU) values within the lesion, and this was considered a prognostic factor for recurrence and was incorporated into their prognostic model. Timmerman et al. [35, 36] used features extracted from cone-beam CT (CBCT) during radiotherapy treatment to validate the previously reported radiomic features for NSCLC outcome prediction [37]. Their works demonstrated that some CT and CBCT radiomic features are interchangeable. Longitudinal CBCT information can be potentially useful clinically in outcome prediction.

Radiomics predictions were not merely applied in the SABR cohort. A study focused on stage III NSCLC patients treated with concurrent chemoradiation proposed that a specific radiomic signature can be used in prediction tumor shrinkage [31]. Specific feature pattern differences between tumor shrinkage or not during chemoradiation can help in treatment decisions for radiotherapy plan adaptation. This study group proposed an ongoing prospective trial (LARTIA Trial, NCT03583723) based on these predictive features, but the results are not available yet.

Similarly, radiation induced lung toxicity can be predicted with similar methods [38–41]. Post SABR CT images were analyzed and certain radiomic features were found to have a dose response relationship [41], suggesting that radiomic features can provide a quantitative measurement of radiation induced lung toxicity after SABR. Traditionally radiation pneumonitis can be predicted with clinical factors including age, combination of systemic treatment, or dosimetric factor, the lung dose exactly. It was shown that incorporating radiomic factors into a prognostic model can better predict radiation pneumonitis [39]. If these radiomic signatures associated with recurrence patterns or toxicity can be validated externally, they might be a good imaging biomarker for radiation treatment.

#### Positron emission tomography (PET)

FDG-PET scan is a common diagnostic tool and is considered standard procedure used in NSCLC staging workup. PET-CT can provide two dimensional information, which includes feature information, which is the same as that of a CT scan, and the biological information, such as maximum standardized uptake value (SUV), metabolic tumor volume (MTV) and total lesion glycolysis (TLG). PET has been utilized in radiotherapy contouring and planning for more than a decade [42]. The idea to target the metabolic tumor volume rather than gross tumor volume is proposed to enhance the therapeutic ratio. Unsupervised machine learning of radiomic features combining functional and texture parameters from FDG-PET scans showed predictive value for treatment response in early stage NSCLC patients post SABR [43]. A high level of pre-treatment SUVmax generally predicted worse local control [44] and possibly worse survival [45]. FDG-PET was also applied in locally advanced lung cancer. Since FDG-PET aims to target the metabolic function of tumors, the question was raised if interim FDG-PET scans can help to better adapt treatment. A prospective single arm study revealed that FDG-PET during chemoradiotherapy is predictive of one-year survival [46]. Individual studies also showed prognostic values of FDG-PET, and PET directed MTV has a better response assessment to radiotherapy than that of the traditionally CT based tumor volume [47]. However, a systemic review failed to show the benefit of interim FDG-PET [48]. The lack of univocal PET parameters among studies might be the major issue. A pilot trial revealed the potential local control benefit of dose escalation based on during-treatment FDG-PET with a fixed risk of radiation induced lung toxitiy (RILT) [49]. RILT, or radiation pneumonitis, is common in thoracic irradiation, and is usually correlated with poor quality of life, impaired lung function, and may be lethal. RILT has been widely studied not only in lung cancer patients but also in esophageal cancer, lymphoma, and breast cancer patients. Ongoing study RTOG 1106, is a PET-directed phase II randomized trial intended to validate this finding. The application of PET scan may be broadened if more solid evidence is available.

FLT-PET, with the tracer fluorothymidine, targets tumor proliferation and is also used in lung cancer and radiotherapy. FLT uptake reduction was observed during and after radiotherapy for lung tumor [50], and was considered more sensitive than FDG-PET scans in comparative studies [51, 52]. With the adjunctive use of FLT-PET, postirradiation changes can be distinguished from tumor recurrence more clearly [53]. Interestingly, correlation with the changes in FLT-PET uptake with a clinical outcome showed that stable FLT uptake was paradoxically associated with longer overall survival and progression-free survival. The results suggested that suppression of tumor cell proliferation might lead to the protection of tumor cell damage from irradiation [51]. Reductionin the bone marrow was also observed during radiotherapy treatment [54, 55], which may be a predictive marker for hematological toxicity.

F-MISO-PET, focused on tissue hypoxia, can help to identify hypoxic areas in tumors for the adaptation of treatment. The uptake of F-MISO was not completely correlated with FDG, suggesting the discordance between tumor glycolysis and hypoxia [56]. During conventional fractionated radiotherapy for NSCLC, a comparison study showed a rapid decrease in FLT uptake, a modest decrease in FDG, and stable uptake of F-MISO [57]. Those results suggested that F-MISO reflected the hypoxia and potentially radioresistant area, not the tumor response during treatment. Adaptive radiotherapy with an escalating dose to the hypoxic area was proposed [58, 59]. Prospective trial based on F-MISO PET is ongoing (RTEP5 trial, NCT01576796).

#### Dosiomics

Dosiomics is a novel idea of radiotherapy emerged in these 2 years. It is an extension from radiomics. The 3D information from the dose distribution in the radiotherapy plan can also be considered as an image for data extraction. In addition to the traditional dose volume histogram (DVH) and logistic normal tissue complication probability (NTCP) model, dosiomics provides a new approach for modeling treatment related toxicity. This approach was proposed in the prostate cancer HYPRO trial to predict gastrointestinal (GI) and genitourinary (GU) toxicities [60], and the results showed that including dosiomic factors can improve prediction performance. Dosiomics has also been applied in thoracic malignancy to predict radiation pneumonitis [61]. Traditionally, dosimetric factors in lung from a DVH analysis were the most commonly used in predicting RILT. The mean lung dose (MLD) and the percentage of lung volume received more than 20 Gy (V20) are the two common parameters. NTCP is a mathematical model to predict toxicity via transforming heterogeneous dose distribution to an equivalent uniform dose. These two approaches were compared with dosiomics factors in the NSCLC radiotherapy cohort. The results demonstrated that the dosiomics outperformed the MLD, V20, and NTCP models in predicting radiation pneumonitis [61]. More investigations are warranted to elucidate the application of dosiomics in NSCLC, its predictive value or its further application in adaptive radiotherapy.

### Panomics biomarkers to improve treatment outcome and reduce toxicities

#### Genetics

Like response to drug varied among every single person with the same disease diagnosis, response and toxicity to radiotherapy also differed according to inherent genetic features. Since the biological basis of radiotherapy is to cause DNA double- strand breaks, genes correlated with DNA repair were reported to enhance radiation effects [62, 63] in in vitro and in vivo models With the generalization of genetic testing tools, several genetic testing models to predict radiotherapy responses have been proposed. The standard approach was to identify the different genetic features between radiosensitive and radioresistant samples, either from cell lines, animal models, or patients. Furthermore, to validate the prognostic models in other cohorts.

The most widely studied was the radiosensitivity index (RSI) proposed by Javier F. Torres-Roca and his colleagues [64]. From cancer cell lines, inherent radiosensitivity was tested by measuring the survival fraction at 2 Gy (SF2). Ten genes were selected to derive the prediction model for the tumor radiotherapy response. This genetic prediction model was validated in breast, esophageal, rectal, head and neck, glioblastoma, pancreas, and prostate malignancies and in brain metastases cohorts [65–72]. Despite the good association achieved in retrospective series, no prospective investigations based on RSI are currently available. This study group further combined the RSI and the linear quadratic model to obtain the genomic-adjusted radiation dose (GARD) [73]. GARD was tested in patients from the Total Cancer Care (TCC) protocol, a prospective, observational database. GARD was shown to independently predict clinical outcome in breast, lung, pancreatic, and glioblastoma cancer patients. For the NSCLC cohort, GARD significantly predicted local control with adjusted surgery, stage, histology, and lymphovascular invasion. Currently there are no clinical trials designed based on GARD.

Implications of genetic testing for radiotherapy were studied recently. The major concern is whether germline mutations will increase radiation toxicity or not. The American Society for Radiation Oncology (ASTRO) recently held a workshop to address this issue [74]. As previously known, bi-allelic pathological mutations at certain DNA repair genes, such as those found in ataxia telangiectasia [75], was considered contraindicated to radiotherapy. However, according to the currently available data, no single copy genetic alteration related to the radiation response will definitely increase radiation related toxicity.

The information for the relationship between somatic mutations in tumors and the radiation response was not sufficient to generate a consensus.

#### Single nucleotide polymorphisms

Single nucleotide polymorphisms (SNPs) were also applied to predict radiotherapy response in cancer. Polymorphisms at genes related to DNA repair were most reported, These include XRCC1, BRCA1, and ERCC1 [76–78]. A polymorphism within the promoter of the TGFβ1 gene is also reported to be associated with radiation sensitivity [77]. SNPs were more widely used in predicting RILT. SNPs at DNA repair or synthesis genes including ATM, RAD51, XRCC family, LIG4, and MTHFR were published in many single institutional studies to predict RILT [79–83]. Other SNPs at inflammatory genes, such as TGFβ1 [84–86], or immune modulated genes, such as CBLB [87], a regulator of T-cell response, were also associated with a higher grade of radiation pneumonitis. The problem for SNPs is the reproducibility and generalization across different patient populations [88]. Yuan X et al. reported that a single nucleotide polymorphism at rs1982073:T869C of the TGFβ1 gene is predictive of a higher grade of RP [86] in Caucasian population. Niu X et al. then showed ethic differences in TGFβ1 gene polymorphisms [84]. The rs1982073:T869C did not predict radiation pneumonitis in the Chinese population. Another SNP in the TGFβ1 gene, the AG/GG genotype of n rs11466345 was associated with a higher risk of RP in Chinese NSCLC patients after thoracic radiotherapy.

#### Epigenetics

Radiation induced DNA methylation changes were found with dose dependent relationship and tissue specificity [89–91]. The most successful clinical application is the methylation status of the O^6^-methylguanine-DNA-methyltransferase (MGMT) promoter, which predicts radiation response and prognosis in glioblastoma [92] Epigenetic control also plays an important role in radiation response in NSCLC. Differential methylation profiles were detected in radiosensitive and resistant NSCLC cell lines. Artificially changed methylation profiles by gene knockdown or knockin also modified the in vitro radiation response [93]. The unmethylated IGFBP-3 promotor was found to be associated with poor response to radiation in NSCLC cell lines and poor outcome for patients who received adjuvant chemoradiotherapy [94] In addition to DNA methylation, non-coding RNA was also identified to have an impact on the tissue response to ionizing irradiation. A global decrease in microRNA (miRNA) levels is a common observation in human cancers, indicating that miRNAs may have function in tumor suppression [95]. The most important pathway was to modify p53 tumor suppression network [95–97]. Another subset of miRNAs involved in DNA repair function, is also important in modulating the radiation response. For example, miR-34a, an important tumor-suppressing microRNA, was tested in vivo to sensitize lung tumor to radiation through RAD51 regulation [98]. Collectively, current evidence suggests that gene polymorphisms or epigenetic profiles may serve as biomarkers to predict radiation induced lung toxicity. The information might not be strong enough to guide radiotherapy decisions.

#### Proteome and metabolome

The systemic effect of radiotherapy at the proteome and metabolome profile is another way to predict treatment related toxicity. Serum inflammatory biomarkers as TGFβ, IL-1, KL-6, IL-6 IL-8, PDGF, TGFα, and TNFα are the most addressed [99]. Some early response changes in the levels of CXCL10 (IP-10), CCL2 (MCP-1), eotaxin, and TIMP-1 were also reported to correlate with RILT [100]. However, no single molecule outperforms others in predicting RILT well. A combination approach is more favored. IL-8 and TGFβ1 combined with mean lung dose was reported to have good predictive values for RILT [85]. In addition to inflammatory markers, mass spectrometry (MS)-based proteomic techniques can be used to identify new biomarkers in the prediction of radiation induced lung fibrosis [101]. Pre-RT C4BPA, VTN, α2M, CO4A and CO5 were some peptides identified with mass based strategies to correlate lung toxicity [102, 103]. Immunological markers were also proposed to predict outcome and toxicity. In an NSCLC SABR cohort, pre-treatment high neutrophil-to-lymphocyte ratio (NLR), platelet-to-lymphocyte ratio (PLR), high neutrophil count and the presence of lymphopenia were associated with poor overall survival. Besides, patients with a higher NLR and low serum albumin level had less symptomatic radiation related toxicities [104].

#### Lung cancer specific driver genes

Lung adenocarcinoma is a unique cancer notable for its driver gene dominant patterns. EGFR activating mutation, ALK translocation, KRAS mutation, and HER2 amplification and mutation are the most common driver gene alterations. EGFR mutation, the most important targetable gene mutation, was also investigated for its impact on radiotherapy response [105, 106]. In basic research, EGFR mutant lung cancer cell lines were reported to have better radiosensitivity in low dose fractionation [107]. EGFR is known to modulate nonhomologous end-joining DNA repair. Impaired EGFR potentially sensitized tumors to radiation [108, 109]. Considering the complexity of lung cancer treatments and interactions between treatment strategies, the influence of EGFR mutations on the radiation response has seldom been proven in clinical studies. Radiation response differences between EGFR mutant and wild type patients is an issue under debate and the clinical data showed inconsistent results. A chemoradiation cohort suggested that EGFR mutant patients have shorter progression free survival than patients with wild-type EGFR [110], while another retrospective study showed better in-field local control for EGFR mutant patients after chemoradiation [111]. The discrepancy between studies might reflect the distinct biological feature of EGFR mutant lung cancer. The failure patterns in clinical series [111–113] showed that EGFR mutant tumors tend to distant metastasize rather than local recurrence. Even if the tumor is more responsive, the overall outcome may not be determined by radiotherapy alone.

KRAS mutations are associated with poor survival in NSCLC without adequate target treatment [114]. They were also reported to correlate with radioresistance, yet the biological basis was not fully understood [114–117]. Downstream pathway molecules of RAS including PI3K and Akt were reported to contribute to radioresistance [116, 118]. Other mechanisms are still under investigation. A subset phenotype of KRAS mutant NSCLC characterized by osteopontin/EGFR-dependent MLCC mitosis-like condensed chromatin (MLCC). It was shown to protect against radiation-induced DNA double-strand breaks and to repress negative regulators, including CRMP1 and BIM in in vitro and in animal models [117]. Clinical data addressing this issue are limited. A proton-based liver SABR phase II study showed that KRAS mutations were associated with worse local control. However, lung cancer patients in this study only accounted for less than 10% of the participants [119]. An early stage NSCLC SABR cohort showed that KRAS is a negative predictor of cancer specific survival [120].

Although less discussed, the response to radiation may be more important in squamous cell carcinoma (SqCC). Generally, SqCC is more sensitive to radiotherapy than adenocarcinoma. Aside from immunotherapy, systemic treatment strategies for SqCC are relatively limited without available target therapy. The lack of effective systemic treatment makes radiotherapy more important in squamous lung cancer management. Abazeed ME et al. [121] found that in lung squamous cell carcinoma cell lines, activation of NFE2L2 and KEAP1, key regulators of the oxidative stress response, is associated with radiation resistance though the transcription factor NRF2. NRF2 is responsible for cell protection including the function of scavenging reactive oxygen species (ROS), which was reported to confer radioresistance [122]. The authors proposed the radiation sensitizing effect of a selective PI3K inhibitor, which negatively regulated NFE2L2 and reduce NRF2 levels. Further clinical studies are warranted to prove this concept.

The radiogenomic approach is becoming more applicable as genetic testing techniques improve. We summarized all biomarkers addressed in this review and categorized the strength in predicting radiosensitivity into three categories as shown in Table 2. Biomarkers with clinical references and independent validation were considered strong predictors (category A) for radiation response or toxicity. Those who had several institutional studies with inconclusive results were characterized as moderate predictors (category B). Biomarkers from single retrospective series or data from cell lines / animal models were considered as weak predictors (category C). Collectively, simple biomarkers like CT radiographic patterns or FDG-PET SUV values were easily to be validated in clinical use. However, the benefit could be small. The CT radiographic information may not provide additional information for adaptive treatment. FDG-PET information was applied successfully in treatment adaptation for Hodgkin lymphoma [123] but failed to show definite clinical benefit in driving clinical judgements in head and neck cancer [124]. Current data generally focus on the predictive and prognostic effects. Guiding the adaptive radiotherapy according to genomic information is scarce and not solid enough to support practice changes in non-small cell lung cancer fields.

**Table 2:** Strength of biomarkers in predicting radiation sensitivity in NSCLC

Types of biomarkers	Outcome predictors	Reference	Strength Category
Panomics			
Genomics	Radiosensitiviy Index (RSI)	[64–72]	A
	Genomic-adjusted radiation dose (GARD)	[73]	B
	Lung adenocarcinoma associated gene: KRAS	[114–117]	B
	Squamous cell carcinoma associated genes: KEAP1, NFE2L2	[121]	C
	DNA repair genes: ATM and other mutated genes: PTEN, RB1, TP53 …	[74, 75]	C
SNPs	DNA repair gene sites: XRCC1, BRCA1, and ERCC1 in predicting radiation response	[76–78]	C
	SNPs in predicting radiation related toxicity DNA repair gene sites: ATM, RAD51, XRCC family, LIG4, MTHFR Inflammatory gene sites: TGFβ1 Immune modulated gene sites: CBLB	[79–83] [84–86, 88] [87]	B B C
Epigenetics	unmethylated IGFBP-3 predicting radiation response	[94]	C
	miRNAs level in predicting radiation response	[95–97]	C
Proteome /Metabolome	Serum inflammatory biomarkers in predicting radiation induced toxicity IL-1, KL-6, IL-6, PDGF, TGFα, TGFβ, IL-8 CXCL10 (IP-10), CCL2 (MCP-1), eotaxin, and TIMP-1	[85, 99] [100]	B B
	Mass spectrometry (MS)-based proteomic markers in predicting lung fibrosis C4BPA, VTN, α2M, CO4A, CO5	[101–103]	B
Immunological markers	Serum markers in predicting SABR outcome neutrophil-to-lymphocyte ratio (NLR), platelet-to-lymphocyte ratio (PLR), high neutrophil count	[104]	C
Radiomics			
CT scan	Post SABR local recurrence prediction: Enlarging opacity at 12 months after SABR Bulging margin; loss of linear margin, air bronchogram loss	[25–27]	A
	Combined radiomic features in predicting post SABR recurrence and radiadiation	[30–37]	B
	Combined radiomic features in predicting radiation pneumonitis	[38–41]	B
PET	FDG-PET: Max SUV, metabolic volume, total lesion glycolysis in predicting treatment response	[43–47]	A
	FLT-PET change in predicting tumor response	[50–53]	B
	F-MISO PET in predicting radioresistant area for dose escalation	[56–59]	B
Dosiomics	Combined 3D dosiomitc information in prediction GI, GU and lung toxicity	[60, 61]	B

Abbreviations: *NSCLC* Non-small cell lung cancer. *CT* Computed tomography. *PET* Positron emission tomography. *SUV* Standard uptake volume. *SABR* Stereotactic ablative radiotherapy. *SNP* Single nucleotide polymorphisms

## Conclusion

Emerging data suggesting that incorporating the information genomic, radiomics, and dosiomics techniques into clinical practice can improve treatment quality and make personalized radiotherapy in NSCLC possible. Basic studies addressing genomic issues are plenty, and this study topic has been studies for more than two decades; however, the application remains in outcome and toxicity predictions. The data based on genomic information have not yet extended its use to guide personalized radiotherapy. In contrast, radiomics and dosiomics are rapidly growing fields in the past 10 years. Adaptive radiotherapy according to radiomics information is more common and some clinical trials are ongoing **(Supplementary Table** **1****)**. The reproducibility might be the major cause of this discrepancy. Genomic guided treatment might be at higher cost and time consuming than radiomics guided treatment. Nevertheless, breast cancer is considered a successful example. A 21-gene recurrence score assay has been established as a standard approach in adjuvant treatment for early breast cancer [125]. With a better integration of clinical, genomic, radiomics and even dosiomics factors, it may be expected that precision radiotherapy will be feasible for the treatment of NSCLC in the near future.

## Supplementary information

**Additional file 1: Supplementary Table 1.** Ongoing or recent completed clinical trials with precision radiotherapy strategies in non-small cell lung cancer

## Abbreviations

NGS: Next generation sequence
NSCLC: Non-small cell lung cancer
SABR: Stereotactic ablative radiotherapy
IMRT: Intensity modulated radiotherapy
EORTC: European organization for research and treatment of cancer
CT: Computed tomography
OAR: Organ at risk)
GTV: Gross tumor volume
AAPM: American association of physicists in medicine
AIP: Average intensity projection images
CBCT: Cone-beam CT
PET: Positron emission tomography
SUV: Standardized uptake value
MTV: Metabolic tumor volume
TLG: Total lesion glycolysis
DVH: Dose volume histogram
NTCP: Normal tissue complication probability
MLD: Mean lung dose
V20: Percentage of volume received more than 20 Gy
SF2: Survival fraction at 2 Gy
GARD: Genomic-adjusted radiation dose
SNPs: Single nucleotide polymorphisms
NLR: Neutrophil-to-lymphocyte ratio
PLR: Platelet-to-lymphocyte ratio

## References

[1] Hodson R (2016). Precision medicine. Nature.

[2] Yang C-Y, Yang JC-H, Yang P-C. Precision management of advanced non–small cell lung cancer. Annu Rev Med. 2020;71:117–36. 10.1146/annurev-med-051718-013524 31986082

[3] Chae YK, Pan AP, Davis AA, Patel SP, Carneiro BA, Kurzrock R, Giles FJ (2017). Path toward precision oncology: review of targeted therapy studies and tools to aid in defining "Actionability" of a molecular lesion and patient management support. Mol Cancer Ther.

[4] Badey A, Barateau A, Delaby N, Fau P, Garcia R, De Crevoisier R, Lisbona A (2019). Overview of adaptive radiotherapy in 2019: From implementation to clinical use. Cancer Radiother.

[5] Timmerman R, Paulus R, Galvin J, Michalski J, Straube W, Bradley J, Fakiris A, Bezjak A, Videtic G, Johnstone D, Fowler J, Gore E, Choy H (2010). Stereotactic body radiation therapy for inoperable early stage lung cancer. Jama.

[6] Curran WJ, Paulus R, Langer CJ, Komaki R, Lee JS, Hauser S, Movsas B, Wasserman T, Rosenthal SA, Gore E, Machtay M, Sause W, Cox JD (2011). Sequential vs. concurrent chemoradiation for stage III non-small cell lung cancer: randomized phase III trial RTOG 9410. J Natl Cancer Inst.

[7] Bradley JD, Paulus R, Komaki R, Masters G, Blumenschein G, Schild S, Bogart J, Hu C, Forster K, Magliocco A, Kavadi V, Garces YI, Narayan S, Iyengar P, Robinson C, Wynn RB, Koprowski C, Meng J, Beitler J, Gaur R, Curran W, Choy H (2015). Standard-dose versus high-dose conformal radiotherapy with concurrent and consolidation carboplatin plus paclitaxel with or without cetuximab for patients with stage IIIA or IIIB non-small-cell lung cancer (RTOG 0617): a randomised, two-by-two factorial phase 3 study. Lancet Oncol.

[8] Alhassani A, Chandra A, Chernew ME (2012). The sources of the SGR "hole". N Engl J Med.

[9] De Ruysscher D, Faivre-Finn C, Le Pechoux C, Peeters S, Belderbos J (2014). High-dose re-irradiation following radical radiotherapy for non-small-cell lung cancer. Lancet Oncol.

[10] Magnuson WJ, Lester-Coll NH, Wu AJ, Yang TJ, Lockney NA, Gerber NK, Beal K, Amini A, Patil T, Kavanagh BD, Camidge DR, Braunstein SE, Boreta LC, Balasubramanian SK, Ahluwalia MS, Rana NG, Attia A, Gettinger SN, Contessa JN, Yu JB, Chiang VL (2017). Management of Brain Metastases in tyrosine kinase inhibitor-Naïve epidermal growth factor receptor-mutant non-small-cell lung Cancer: a retrospective multi-institutional analysis. J Clin Oncol.

[11] Iyengar P, Wardak Z, Gerber DE, Tumati V, Ahn C, Hughes RS, Dowell JE, Cheedella N, Nedzi L, Westover KD, Pulipparacharuvil S, Choy H, Timmerman RD (2018). Consolidative radiotherapy for limited metastatic non-small-cell lung Cancer: a phase 2 randomized clinical trial. JAMA Oncol.

[12] Petty WJ, Urbanic JJ, Ahmed T, Hughes R, Levine B, Rusthoven K, Papagikos M, Ruiz JR, Lally BE, Chan M, Clark H, D'Agostino RB, Blackstock AW (2018). Long-term outcomes of a phase 2 trial of chemotherapy with consolidative radiation therapy for Oligometastatic non-small cell lung Cancer. Int J Radiat Oncol Biol Phys.

[13] Caudell JJ, Torres-Roca JF, Gillies RJ, Enderling H, Kim S, Rishi A, Moros EG, Harrison LB (2017). The future of personalised radiotherapy for head and neck cancer. Lancet Oncology.

[14] Bernier J (2017). Precision medicine for early breast cancer radiotherapy: opening up new horizons?. Crit Rev Oncol Hematol.

[15] Zhang MX, Li J, Shen GP, Zou X, Xu JJ, Jiang R, You R, Hua YJ, Sun Y, Ma J, Hong MH, Chen MY (2015). Intensity-modulated radiotherapy prolongs the survival of patients with nasopharyngeal carcinoma compared with conventional two-dimensional radiotherapy: a 10-year experience with a large cohort and long follow-up. Eur J Cancer.

[16] Paganetti H, Jiang H, Parodi K, Slopsema R, Engelsman M (2008). Clinical implementation of full Monte Carlo dose calculation in proton beam therapy. Phys Med Biol.

[17] Kamada T, Tsujii H, Blakely EA, Debus J, De Neve W, Durante M, Jakel O, Mayer R, Orecchia R, Potter R, Vatnitsky S, Chu WT (2015). Carbon ion radiotherapy in Japan: an assessment of 20 years of clinical experience. Lancet Oncol.

[18] De Ruysscher D, Faivre-Finn C, Moeller D, Nestle U, Hurkmans CW, Le Pechoux C, Belderbos J, Guckenberger M, Senan S (2017). European Organization for Research and Treatment of Cancer (EORTC) recommendations for planning and delivery of high-dose, high precision radiotherapy for lung cancer. Radiother Oncol.

[19] Wilson R, Devaraj A (2017). Radiomics of pulmonary nodules and lung cancer. Transl Lung Cancer Res.

[20] Kumar V, Gu Y, Basu S, Berglund A, Eschrich SA, Schabath MB, Forster K, Aerts HJ, Dekker A, Fenstermacher D, Goldgof DB, Hall LO, Lambin P, Balagurunathan Y, Gatenby RA, Gillies RJ (2012). Radiomics: the process and the challenges. Magn Reson Imaging.

[21] Chen B, Zhang R, Gan Y, Yang L, Li W (2017). Development and clinical application of radiomics in lung cancer. Radiat Oncol.

[22] Lambin P, Leijenaar RTH, Deist TM, Peerlings J, de Jong EEC, van Timmeren J, Sanduleanu S, Larue R, Even AJG, Jochems A, van Wijk Y, Woodruff H, van Soest J, Lustberg T, Roelofs E, van Elmpt W, Dekker A, Mottaghy FM, Wildberger JE, Walsh S (2017). Radiomics: the bridge between medical imaging and personalized medicine. Nat Rev Clin Oncol.

[23] Yang J, Veeraraghavan H, Armato SG, Farahani K, Kirby JS, Kalpathy-Kramer J, van Elmpt W, Dekker A, Han X, Feng X, Aljabar P, Oliveira B, van der Heyden B, Zamdborg L, Lam D, Gooding M, Sharp GC (2018). Autosegmentation for thoracic radiation treatment planning: a grand challenge at AAPM 2017. Med Phys.

[24] Parmar C, Rios VE, Leijenaar R, Jermoumi M, Carvalho S, Mak RH, Mitra S, Shankar BU, Kikinis R, Haibe-Kains B, Lambin P, Aerts HJ (2014). Robust Radiomics feature quantification using semiautomatic volumetric segmentation. PLoS One.

[25] Huang K, Dahele M, Senan S, Guckenberger M, Rodrigues GB, Ward A, Boldt RG, Palma DA (2012). Radiographic changes after lung stereotactic ablative radiotherapy (SABR)--can we distinguish recurrence from fibrosis? A systematic review of the literature. Radiother Oncol.

[26] Huang K, Senthi S, Palma DA, Spoelstra FO, Warner A, Slotman BJ, Senan S (2013). High-risk CT features for detection of local recurrence after stereotactic ablative radiotherapy for lung cancer. Radiother Oncol.

[27] Peulen H, Mantel F, Guckenberger M, Belderbos J, Werner-Wasik M, Hope A, Giuliani M, Grills I, Sonke JJ (2016). Validation of high-risk computed tomography features for detection of local recurrence after stereotactic body radiation therapy for early-stage non-small cell lung Cancer. Int J Radiat Oncol Biol Phys.

[28] Huynh E, Coroller TP, Narayan V, Agrawal V, Romano J, Franco I, Parmar C, Hou Y, Mak RH, Aerts HJ (2017). Associations of Radiomic data extracted from static and respiratory-gated CT scans with disease recurrence in lung Cancer patients treated with SBRT. PLoS One.

[29] Mattonen SA, Palma DA, Johnson C, Louie AV, Landis M, Rodrigues G, Chan I, Etemad-Rezai R, Yeung TP, Senan S, Ward AD (2016). Detection of local Cancer recurrence after stereotactic ablative radiation therapy for lung Cancer: physician performance versus Radiomic assessment. Int J Radiat Oncol Biol Phys.

[30] Li Q, Kim J, Balagurunathan Y, Liu Y, Latifi K, Stringfield O, Garcia A, Moros EG, Dilling TJ, Schabath MB, Ye Z, Gillies RJ (2017). Imaging features from pretreatment CT scans are associated with clinical outcomes in nonsmall-cell lung cancer patients treated with stereotactic body radiotherapy. Med Phys.

[31] Ramella S, Fiore M, Greco C, Cordelli E, Sicilia R, Merone M, Molfese E, Miele M, Cornacchione P, Ippolito E, Iannello G, D'Angelillo RM, Soda P (2018). A radiomic approach for adaptive radiotherapy in non-small cell lung cancer patients. PLoS One.

[32] Shi L, He Y, Yuan Z, Benedict S, Valicenti R, Qiu J, Rong Y (2018). Radiomics for response and outcome assessment for non-small cell lung Cancer. Technol Cancer Res Treat.

[33] Zhang Y, Oikonomou A, Wong A, Haider MA, Khalvati F (2017). Radiomics-based prognosis analysis for non-small cell lung Cancer. Sci Rep.

[34] Huynh E, Coroller TP, Narayan V, Agrawal V, Hou Y, Romano J, Franco I, Mak RH, Aerts HJ (2016). CT-based radiomic analysis of stereotactic body radiation therapy patients with lung cancer. Radiother Oncol.

[35] van Timmeren JE, Leijenaar RTH, van Elmpt W, Reymen B, Oberije C, Monshouwer R, Bussink J, Brink C, Hansen O, Lambin P (2017). Survival prediction of non-small cell lung cancer patients using radiomics analyses of cone-beam CT images. Radiother Oncol.

[36] van Timmeren JE, van Elmpt W, Leijenaar RTH, Reymen B, Monshouwer R, Bussink J, Paelinck L, Bogaert E, De Wagter C, Elhaseen E, Lievens Y, Hansen O, Brink C, Lambin P (2019). Longitudinal radiomics of cone-beam CT images from non-small cell lung cancer patients: evaluation of the added prognostic value for overall survival and locoregional recurrence. Radiother Oncol.

[37] Aerts HJ, Velazquez ER, Leijenaar RT, Parmar C, Grossmann P, Carvalho S, Bussink J, Monshouwer R, Haibe-Kains B, Rietveld D, Hoebers F, Rietbergen MM, Leemans CR, Dekker A, Quackenbush J, Gillies RJ, Lambin P (2014). Decoding tumour phenotype by noninvasive imaging using a quantitative radiomics approach. Nat Commun.

[38] Bousabarah K, Temming S, Hoevels M, Borggrefe J, Baus WW, Ruess D, Visser-Vandewalle V, Ruge M, Kocher M, Treuer H. Radiomic analysis of planning computed tomograms for predicting radiation-induced lung injury and outcome in lung cancer patients treated with robotic stereotactic body radiation therapy. Strahlenther Onkol. 2019. 10.1007/s00066-019-01452-7 30874846

[39] Krafft SP, Rao A, Stingo F, Briere TM, Court LE, Liao Z, Martel MK (2018). The utility of quantitative CT radiomics features for improved prediction of radiation pneumonitis. Med Phys.

[40] Luo Y, McShan DL, Matuszak MM, Ray D, Lawrence TS, Jolly S, Kong FM, Ten Haken RK, El Naqa I. A multiobjective Bayesian networks approach for joint prediction of tumor local control and radiation pneumonitis in nonsmall-cell lung cancer (NSCLC) for response-adapted radiotherapy. Med Phys. 2018. 10.1002/mp.13029 PMC6279602 29862533

[41] Moran A, Daly ME, Yip SSF, Yamamoto T (2017). Radiomics-based assessment of radiation-induced lung injury after stereotactic body radiotherapy. Clin Lung Cancer.

[42] Grills IS, Yan D, Black QC, Wong CY, Martinez AA, Kestin LL (2007). Clinical implications of defining the gross tumor volume with combination of CT and 18FDG-positron emission tomography in non-small-cell lung cancer. Int J Radiat Oncol Biol Phys.

[43] Li H, Galperin-Aizenberg M, Pryma D, Simone CB, Fan Y (2018). Unsupervised machine learning of radiomic features for predicting treatment response and overall survival of early stage non-small cell lung cancer patients treated with stereotactic body radiation therapy. Radiother Oncol.

[44] Kandi M, Hoffmann L, Sloth MD, Schmidt HH, Knap MM, Khalil AA (2018). Local failure after radical radiotherapy of non-small cell lung cancer in relation to the planning FDG-PET/CT. Acta Oncol.

[45] Dong M, Liu J, Sun X, Xing L (2017). Prognositc significance of SUVmax on pretreatment (18) F-FDG PET/CT in early-stage non-small cell lung cancer treated with stereotactic body radiotherapy: a meta-analysis. J Med Imaging Radiat Oncol.

[46] Vera P, Mezzani-Saillard S, Edet-Sanson A, Menard JF, Modzelewski R, Thureau S, Meyer ME, Jalali K, Bardet S, Lerouge D, Houzard C, Mornex F, Olivier P, Faure G, Rousseau C, Mahe MA, Gomez P, Brenot-Rossi I, Salem N, Dubray B (2014). FDG PET during radiochemotherapy is predictive of outcome at 1 year in non-small-cell lung cancer patients: a prospective multicentre study (RTEP2). Eur J Nucl Med Mol Imaging.

[47] Mahasittiwat P, Yuan S, Xie C, Ritter T, Cao Y, Ten Haken RK, Kong FM (2013). Metabolic tumor volume on PET reduced more than Gross tumor volume on CT during radiotherapy in patients with non-small cell lung Cancer treated with 3DCRT or SBRT. J Radiat Oncol.

[48] Cremonesi M, Gilardi L, Ferrari ME, Piperno G, Travaini LL, Timmerman R, Botta F, Baroni G, Grana CM, Ronchi S, Ciardo D, Jereczek-Fossa BA, Garibaldi C, Orecchia R (2017). Role of interim (18)F-FDG-PET/CT for the early prediction of clinical outcomes of non-small cell lung Cancer (NSCLC) during radiotherapy or chemo-radiotherapy. A systematic review. Eur J Nucl Med Mol Imaging.

[49] Kong FM, Ten Haken RK, Schipper M, Frey KA, Hayman J, Gross M, Ramnath N, Hassan KA, Matuszak M, Ritter T, Bi N, Wang W, Orringer M, Cease KB, Lawrence TS, Kalemkerian GP (2017). Effect of Midtreatment PET/CT-adapted radiation therapy with concurrent chemotherapy in patients with locally advanced non-small-cell lung Cancer: a phase 2 clinical trial. JAMA Oncol.

[50] Everitt S, Hicks RJ, Ball D, Kron T, Schneider-Kolsky M, Walter T, Binns D, Mac MM (2009). Imaging cellular proliferation during chemo-radiotherapy: a pilot study of serial 18F-FLT positron emission tomography/computed tomography imaging for non-small-cell lung cancer. Int J Radiat Oncol Biol Phys.

[51] Everitt S, Ball D, Hicks RJ, Callahan J, Plumridge N, Trinh J, Herschtal A, Kron T, Mac MM (2017). Prospective study of serial imaging comparing Fluorodeoxyglucose positron emission tomography (PET) and Fluorothymidine PET during radical Chemoradiation for non-small cell lung Cancer: reduction of detectable proliferation associated with worse survival. Int J Radiat Oncol Biol Phys.

[52] Everitt SJ, Ball DL, Hicks RJ, Callahan J, Plumridge N, Collins M, Herschtal A, Binns D, Kron T, Schneider M, MacManus M (2014). Differential (18)F-FDG and (18)F-FLT uptake on serial PET/CT imaging before and during definitive Chemoradiation for non-small cell lung Cancer. J Nucl Med.

[53] Hiniker SM, Sodji Q, Quon A, Gutkin PM, Arksey N, Graves EE, Chin FT, Maxim PG, Diehn M, Loo BW (2019). FLT-PET-CT for the detection of disease recurrence after stereotactic ablative radiotherapy or Hyperfractionation for thoracic malignancy: a prospective pilot study. Front Oncol.

[54] Campbell BA, Callahan J, Bressel M, Simoens N, Everitt S, Hofman MS, Hicks RJ, Burbury K, MacManus M (2015). Distribution atlas of proliferating bone marrow in non-small cell lung Cancer patients measured by FLT-PET/CT imaging, with potential applicability in radiation therapy planning. Int J Radiat Oncol Biol Phys.

[55] Leimgruber A, Moller A, Everitt SJ, Chabrot M, Ball DL, Solomon B, MacManus M, Hicks RJ (2014). Effect of platinum-based Chemoradiotherapy on cellular proliferation in bone marrow and spleen, estimated by (18)F-FLT PET/CT in patients with locally advanced non-small cell lung Cancer. J Nucl Med.

[56] Sachpekidis C, Thieke C, Askoxylakis V, Nicolay NH, Huber PE, Thomas M, Dimitrakopoulou G, Debus J, Haberkorn U, Dimitrakopoulou-Strauss A (2015). Combined use of (18)F-FDG and (18)F-FMISO in unresectable non-small cell lung cancer patients planned for radiotherapy: a dynamic PET/CT study. Am J Nucl Med Mol Imaging.

[57] Vera P, Bohn P, Edet-Sanson A, Salles A, Hapdey S, Gardin I, Menard JF, Modzelewski R, Thiberville L, Dubray B (2011). Simultaneous positron emission tomography (PET) assessment of metabolism with (1)(8)F-fluoro-2-deoxy-d-glucose (FDG), proliferation with (1)(8)F-fluoro-thymidine (FLT), and hypoxia with (1)(8)fluoro-misonidazole (F-miso) before and during radiotherapy in patients with non-small-cell lung cancer (NSCLC): a pilot study. Radiother Oncol.

[58] Thureau S, Dubray B, Modzelewski R, Bohn P, Hapdey S, Vincent S, Anger E, Gensanne D, Pirault N, Pierrick G, Vera P (2018). FDG and FMISO PET-guided dose escalation with intensity-modulated radiotherapy in lung cancer. Radiat Oncol.

[59] Tachibana I, Nishimura Y, Shibata T, Kanamori S, Nakamatsu K, Koike R, Nishikawa T, Ishikawa K, Tamura M, Hosono M (2013). A prospective clinical trial of tumor hypoxia imaging with 18F-fluoromisonidazole positron emission tomography and computed tomography (F-MISO PET/CT) before and during radiation therapy. J Radiat Res.

[60] Rossi L, Bijman R, Schillemans W, Aluwini S, Cavedon C, Witte M, Incrocci L, Heijmen B (2018). Texture analysis of 3D dose distributions for predictive modelling of toxicity rates in radiotherapy. Radiother Oncol.

[61] Liang B, Yan H, Tian Y, Chen X, Yan L, Zhang T, Zhou Z, Wang L, Dai J (2019). Dosiomics: extracting 3D spatial features from dose distribution to predict incidence of radiation pneumonitis. Front Oncol.

[62] Moullan N, Cox DG, Angele S, Romestaing P, Gerard JP, Hall J (2003). Polymorphisms in the DNA repair gene XRCC1, breast cancer risk, and response to radiotherapy. Cancer Epidemiol Biomark Prev.

[63] Thoms J, Bristow RG (2010). DNA repair targeting and radiotherapy: a focus on the therapeutic ratio. Semin Radiat Oncol.

[64] Torres-Roca JF, Eschrich S, Zhao H, Bloom G, Sung J, McCarthy S, Cantor AB, Scuto A, Li C, Zhang S, Jove R, Yeatman T (2005). Prediction of radiation sensitivity using a gene expression classifier. Cancer Res.

[65] Ahmed KA, Berglund AE, Welsh EA, Naghavi AO, Kim Y, Yu M, Robinson TJ, Eschrich SA, Johnstone PAS, Torres-Roca JF (2017). The radiosensitivity of brain metastases based upon primary histology utilizing a multigene index of tumor radiosensitivity. Neuro-Oncology.

[66] Ahmed KA, Caudell JJ, El-Haddad G, Berglund AE, Welsh EA, Yue B, Hoffe SE, Naghavi AO, Abuodeh YA, Frakes JM, Eschrich SA, Torres-Roca JF (2016). Radiosensitivity differences between liver metastases based on primary histology suggest implications for clinical outcomes after stereotactic body radiation therapy. Int J Radiat Oncol Biol Phys.

[67] Ahmed KA, Fulp WJ, Berglund AE, Hoffe SE, Dilling TJ, Eschrich SA, Shridhar R, Torres-Roca JF (2015). Differences between Colon Cancer primaries and metastases using a molecular assay for tumor radiation sensitivity suggest implications for potential Oligometastatic SBRT patient selection. Int J Radiat Oncol Biol Phys.

[68] Ahmed KA, Grass GD, Orman AG, Liveringhouse C, Montejo ME, Soliman HH, Han HS, Czerniecki BJ, Torres-Roca JF, Diaz R (2018). Personalizing radiation treatment delivery in the Management of Breast Cancer. Int J Breast Cancer.

[69] Ahmed KA, Scott JG, Arrington JA, Naghavi AO, Grass GD, Perez BA, Caudell JJ, Berglund AE, Welsh EA, Eschrich SA, Dilling TJ, Torres-Roca JF (2018). Radiosensitivity of lung metastases by primary histology and implications for stereotactic body radiation therapy using the Genomically adjusted radiation dose. J Thorac Oncol.

[70] Eschrich SA, Fulp WJ, Pawitan Y, Foekens JA, Smid M, Martens JW, Echevarria M, Kamath V, Lee JH, Harris EE, Bergh J, Torres-Roca JF (2012). Validation of a radiosensitivity molecular signature in breast cancer. Clin Cancer Res.

[71] Eschrich SA, Pramana J, Zhang H, Zhao H, Boulware D, Lee JH, Bloom G, Rocha-Lima C, Kelley S, Calvin DP, Yeatman TJ, Begg AC, Torres-Roca JF (2009). A gene expression model of intrinsic tumor radiosensitivity: prediction of response and prognosis after chemoradiation. Int J Radiat Oncol Biol Phys.

[72] Torres-Roca JF, Fulp WJ, Caudell JJ, Servant N, Bollet MA, van de Vijver M, Naghavi AO, Harris EE, Eschrich SA (2015). Integration of a Radiosensitivity molecular signature into the assessment of local recurrence risk in breast Cancer. Int J Radiat Oncol Biol Phys.

[73] Scott JG, Berglund A, Schell MJ, Mihaylov I, Fulp WJ, Yue B, Welsh E, Caudell JJ, Ahmed K, Strom TS, Mellon E, Venkat P, Johnstone P, Foekens J, Lee J, Moros E, Dalton WS, Eschrich SA, McLeod H, Harrison LB, Torres-Roca JF (2017). A genome-based model for adjusting radiotherapy dose (GARD): a retrospective, cohort-based study. Lancet Oncol.

[74] Bergom C, West CM, Higginson DS, Abazeed ME, Arun B, Bentzen SM, Bernstein JL, Evans JD, Gerber NK, Kerns SL, Keen J, Litton JK, Reiner AS, Riaz N, Rosenstein BS, Sawakuchi GO, Shaitelman SF, Powell SN, Woodward WA. The implications of genetic testing on radiotherapy decisions: a guide for radiation oncologists. Int J Radiat Oncol Biol Phys. 2019;105(4):698–712. 10.1016/j.ijrobp.2019.07.026 PMC11913060 31381960

[75] Taylor AM, Groom A, Byrd PJ (2004). Ataxia-telangiectasia-like disorder (ATLD)-its clinical presentation and molecular basis. DNA Repair (Amst).

[76] Kelsey CR, Jackson IL, Langdon S, Owzar K, Hubbs J, Vujaskovic Z, Das S, Marks LB (2013). Analysis of single nucleotide polymorphisms and radiation sensitivity of the lung assessed with an objective radiologic endpoin. Clin Lung Cancer.

[77] Kelsey CR, Jackson L, Langdon S, Owzar K, Hubbs J, Vujaskovic Z, Das S, Marks LB (2012). A polymorphism within the promoter of the TGFbeta1 gene is associated with radiation sensitivity using an objective radiologic endpoint. Int J Radiat Oncol Biol Phys.

[78] Su D, Ma S, Liu P, Jiang Z, Lv W, Zhang Y, Deng Q, Smith S, Yu H (2007). Genetic polymorphisms and treatment response in advanced non-small cell lung cancer. Lung Cancer.

[79] Wen J, Liu H, Wang L, Wang X, Gu N, Liu Z, Xu T, Gomez DR, Komaki R, Liao Z, Wei Q (2018). Potentially functional variants of ATG16L2 predict radiation pneumonitis and outcomes in patients with non-small cell lung Cancer after definitive radiotherapy. J Thorac Oncol.

[80] Wen J, Liu H, Wang Q, Liu Z, Li Y, Xiong H, Xu T, Li P, Wang LE, Gomez DR, Mohan R, Komaki R, Liao Z, Wei Q (2014). Genetic variants of the LIN28B gene predict severe radiation pneumonitis in patients with non-small cell lung cancer treated with definitive radiation therapy. Eur J Cancer.

[81] Xiong H, Liao Z, Liu Z, Xu T, Wang Q, Liu H, Komaki R, Gomez D, Wang LE, Wei Q (2013). ATM polymorphisms predict severe radiation pneumonitis in patients with non-small cell lung cancer treated with definitive radiation therapy. Int J Radiat Oncol Biol Phys.

[82] Yang J, Xu T, Gomez DR, Yuan X, Nguyen QN, Jeter M, Song Y, Hahn S, Liao Z (2017). Polymorphisms in BMP2/BMP4, with estimates of mean lung dose, predict radiation pneumonitis among patients receiving definitive radiotherapy for non-small cell lung cancer. Oncotarget.

[83] Yin M, Liao Z, Liu Z, Wang LE, Gomez D, Komaki R, Wei Q (2011). Functional polymorphisms of base excision repair genes XRCC1 and APEX1 predict risk of radiation pneumonitis in patients with non-small cell lung cancer treated with definitive radiation therapy. Int J Radiat Oncol Biol Phys.

[84] Niu X, Li H, Chen Z, Liu Y, Kan M, Zhou D, Li Z, Ye X, Shen S, Lv C, Lu S (2012). A study of ethnic differences in TGFbeta1 gene polymorphisms and effects on the risk of radiation pneumonitis in non-small-cell lung cancer. J Thorac Oncol.

[85] Wang S, Campbell J, Stenmark MH, Zhao J, Stanton P, Matuszak MM, Ten Haken RK, Kong FS (2017). Plasma levels of IL-8 and TGF-beta1 predict radiation-induced lung toxicity in non-small cell lung Cancer: a validation study. Int J Radiat Oncol Biol Phys.

[86] Yuan X, Liao Z, Liu Z, Wang LE, Tucker SL, Mao L, Wang XS, Martel M, Komaki R, Cox JD, Milas L, Wei Q (2009). Single nucleotide polymorphism at rs1982073:T869C of the TGFbeta 1 gene is associated with the risk of radiation pneumonitis in patients with non-small-cell lung cancer treated with definitive radiotherapy. J Clin Oncol.

[87] Li P, Wang X, Liu Z, Liu H, Xu T, Wang H, Gomez DR, Nguyen QN, Wang LE, Teng Y, Song Y, Komaki R, Welsh JW, Wei Q, Liao Z (2016). Single nucleotide polymorphisms in CBLB, a regulator of T-cell response, predict radiation pneumonitis and outcomes after definitive radiotherapy for non-small-cell lung cancer. Clin Lung Cancer.

[88] Huang Q, Xie F, Ouyang X (2015). Predictive SNPs for radiation-induced damage in lung cancer patients with radiotherapy: a potential strategy to individualize treatment. Int J Biol Markers.

[89] Pogribny I, Raiche J, Slovack M, Kovalchuk O (2004). Dose-dependence, sex- and tissue-specificity, and persistence of radiation-induced genomic DNA methylation changes. Biochem Biophys Res Commun.

[90] Miousse IR, Kutanzi KR, Koturbash I (2017). Effects of ionizing radiation on DNA methylation: from experimental biology to clinical applications. Int J Radiat Biol.

[91] Koturbash I, Griffin RJ (2019). Harnessing epigenetics and metabolism to modulate tissue response to radiotherapy. Int J Radiat Biol.

[92] Rivera AL, Pelloski CE, Gilbert MR, Colman H, De La Cruz C, Sulman EP, Bekele BN, Aldape KD (2010). MGMT promoter methylation is predictive of response to radiotherapy and prognostic in the absence of adjuvant alkylating chemotherapy for glioblastoma. Neuro-Oncology.

[93] Kim EH, Park AK, Dong SM, Ahn JH, Park WY (2010). Global analysis of CpG methylation reveals epigenetic control of the radiosensitivity in lung cancer cell lines. Oncogene.

[94] Pernia O, Belda-Iniesta C, Pulido V, Cortes-Sempere M, Rodriguez C, Vera O, Soto J, Jimenez J, Taus A, Rojo F, Arriola E, Rovira A, Albanell J, Macias MT, de Castro J, Perona R, Ibanez de Caceres I (2014). Methylation status of IGFBP-3 as a useful clinical tool for deciding on a concomitant radiotherapy. Epigenetics.

[95] He L, He X, Lim LP, de Stanchina E, Xuan Z, Liang Y, Xue W, Zender L, Magnus J, Ridzon D, Jackson AL, Linsley PS, Chen C, Lowe SW, Cleary MA, Hannon GJ (2007). A microRNA component of the p53 tumour suppressor network. Nature.

[96] Chakraborty S, Mazumdar M, Mukherjee S, Bhattacharjee P, Adhikary A, Manna A, Chakraborty S, Khan P, Sen A, Das T (2014). Restoration of p53/miR-34a regulatory axis decreases survival advantage and ensures Bax-dependent apoptosis of non-small cell lung carcinoma cells. FEBS Lett.

[97] Rahman M, Lovat F, Romano G, Calore F, Acunzo M, Bell EH, Nana-Sinkam P (2014). miR-15b/16–2 regulates factors that promote p53 phosphorylation and augments the DNA damage response following radiation in the lung. J Biol Chem.

[98] Cortez MA, Valdecanas D, Niknam S, Peltier HJ, Diao L, Giri U, Komaki R, Calin GA, Gomez DR, Chang JY, Heymach JV, Bader AG, Welsh JW (2015). In vivo delivery of miR-34a sensitizes lung tumors to radiation through RAD51 regulation. Mol Ther Nucleic Acids.

[99] Kong FM, Ao X, Wang L, Lawrence TS (2008). The use of blood biomarkers to predict radiation lung toxicity: a potential strategy to individualize thoracic radiation therapy. Cancer Control.

[100] Sprung CN, Forrester HB, Siva S, Martin OA (2015). Immunological markers that predict radiation toxicity. Cancer Lett.

[101] Jelonek K, Pietrowska M, Widlak P (2017). Systemic effects of ionizing radiation at the proteome and metabolome levels in the blood of cancer patients treated with radiotherapy: the influence of inflammation and radiation toxicity. Int J Radiat Biol.

[102] Cai XW, Shedden KA, Yuan SH, Davis MA, Xu LY, Xie CY, Fu XL, Lawrence TS, Lubman DM, Kong FM (2011). Baseline plasma proteomic analysis to identify biomarkers that predict radiation-induced lung toxicity in patients receiving radiation for non-small cell lung cancer. J Thorac Oncol.

[103] Oh JH, Craft JM, Townsend R, Deasy JO, Bradley JD, El Naqa I (2011). A bioinformatics approach for biomarker identification in radiation-induced lung inflammation from limited proteomics data. J Proteome Res.

[104] Shaverdian N, Veruttipong D, Wang J, Schaue D, Kupelian P, Lee P (2016). Pretreatment immune parameters predict for overall survival and toxicity in early-stage non-small-cell lung Cancer patients treated with stereotactic body radiation therapy. Clin Lung Cancer.

[105] Choi EJ, Ryu YK, Kim SY, Wu HG, Kim JS, Kim IH, Kim IA (2010). Targeting epidermal growth factor receptor-associated signaling pathways in non-small cell lung cancer cells: implication in radiation response. Mol Cancer Res.

[106] Das AK, Chen BP, Story MD, Sato M, Minna JD, Chen DJ, Nirodi CS (2007). Somatic mutations in the tyrosine kinase domain of epidermal growth factor receptor (EGFR) abrogate EGFR-mediated radioprotection in non-small cell lung carcinoma. Cancer Res.

[107] Das AK, Sato M, Story MD, Peyton M, Graves R, Redpath S, Girard L, Gazdar AF, Shay JW, Minna JD, Nirodi CS (2006). Non-small-cell lung cancers with kinase domain mutations in the epidermal growth factor receptor are sensitive to ionizing radiation. Cancer Res.

[108] Chen DJ, Nirodi CS (2007). The epidermal growth factor receptor: a role in repair of radiation-induced DNA damage. Clin Cancer Res.

[109] Mukherjee B, Choy H, Nirodi C, Burma S (2010). Targeting nonhomologous end-joining through epidermal growth factor receptor inhibition: rationale and strategies for radiosensitization. Semin Radiat Oncol.

[110] Wang N, Wang L, Meng X, Wang J, Zhu L, Liu C, Li S, Zheng L, Yang Z, Xing L, Yu J (2019). Osimertinib (AZD9291) increases radiosensitivity in EGFR T790M nonsmall cell lung cancer. Oncol Rep.

[111] Lim YJ, Chang JH, Kim HJ, Keam B, Kim TM, Kim DW, Paeng JC, Kang KW, Chung JK, Jeon YK, Chung DH, Wu HG (2017). Superior treatment response and in-field tumor control in epidermal growth factor receptor-mutant genotype of stage III nonsquamous non-small cell lung Cancer undergoing definitive concurrent Chemoradiotherapy. Clin Lung Cancer.

[112] Johung KL, Yao X, Li F, Yu JB, Gettinger SN, Goldberg S, Decker RH, Hess JA, Chiang VL, Contessa JN (2013). A clinical model for identifying radiosensitive tumor genotypes in non-small cell lung cancer. Clin Cancer Res.

[113] Tanaka K, Hida T, Oya Y, Oguri T, Yoshida T, Shimizu J, Horio Y, Hata A, Kaji R, Fujita S, Sekido Y, Kodaira T, Kokubo M, Katakami N, Yatabe Y (2015). EGFR mutation impact on definitive concurrent Chemoradiation therapy for inoperable stage III adenocarcinoma. J Thorac Oncol.

[114] Ferrer I, Zugazagoitia J, Herbertz S, John W, Paz-Ares L, Schmid-Bindert G (2018). KRAS-mutant non-small cell lung cancer: from biology to therapy. Lung Cancer.

[115] Gupta A.K., Bakanauskas V.J., Cerniglia G.J., Cheng Y., Bernhard E.J., Muschel R.J. and McKenna W.G. The Ras radiation resistance pathway. Cancer Res 61(10):4278–4282, 2001. 11358856

[116] McKenna WG, Muschel RJ, Gupta AK, Hahn SM, Bernhard EJ (2003). The RAS signal transduction pathway and its role in radiation sensitivity. Oncogene.

[117] Wang M, Han J, Marcar L, Black J, Liu Q, Li X, Nagulapalli K, Sequist LV, Mak RH, Benes CH, Hong TS, Gurtner K, Krause M, Baumann M, Kang JX, Whetstine JR, Willers H (2017). Radiation resistance in KRAS-mutated lung Cancer is enabled by stem-like properties mediated by an Osteopontin-EGFR pathway. Cancer Res.

[118] Brunner TB, Hahn SM, McKenna WG, Bernhard EJ (2004). Radiation sensitization by inhibition of activated Ras. Strahlenther Onkol.

[119] Hong TS, Wo JY, Borger DR, et al. Phase II study of proton-based stereotactic body radiation therapy for liver metastases: importance of tumor genotype. J Natl Cancer Inst. 2017;109(9):10.1093/jnci/djx031. 10.1093/jnci/djx031. 10.1093/jnci/djx031 28954285

[120] Mak RH, Hermann G, Lewis JH, Aerts HJ, Baldini EH, Chen AB, Colson YL, Hacker FH, Kozono D, Wee JO, Chen YH, Catalano PJ, Wong KK, Sher DJ (2015). Outcomes by tumor histology and KRAS mutation status after lung stereotactic body radiation therapy for early-stage non-small-cell lung cancer. Clin Lung Cancer.

[121] Abazeed ME, Adams DJ, Hurov KE, Tamayo P, Creighton CJ, Sonkin D, Giacomelli AO, Du C, Fries DF, Wong KK, Mesirov JP, Loeffler JS, Schreiber SL, Hammerman PS, Meyerson M (2013). Integrative radiogenomic profiling of squamous cell lung cancer. Cancer Res.

[122] Singh A, Bodas M, Wakabayashi N, Bunz F, Biswal S (2010). Gain of Nrf2 function in non-small-cell lung cancer cells confers radioresistance. Antioxid Redox Signal.

[123] Barrington SF, Kirkwood AA, Franceschetto A, Fulham MJ, Roberts TH, Almquist H, Brun E, Hjorthaug K, Viney ZN, Pike LC, Federico M, Luminari S, Radford J, Trotman J, Fosså A, Berkahn L, Molin D, D'Amore F, Sinclair DA, Smith P, O'Doherty MJ, Stevens L, Johnson PW (2016). PET-CT for staging and early response: results from the response-adapted therapy in advanced Hodgkin lymphoma study. Blood.

[124] Hamming-Vrieze O, Navran A, Al-Mamgani A, Vogel WV (2018). Biological PET-guided adaptive radiotherapy for dose escalation in head and neck cancer: a systematic review. Q J Nucl Med Mol Imaging.

[125] Levine MN, Julian JA, Bedard PL, Eisen A, Trudeau ME, Higgins B, Bordeleau L, Pritchard KI (2016). Prospective evaluation of the 21-gene recurrence score assay for breast Cancer decision-making in Ontario. J Clin Oncol.

