# Supplementary material for: Precision radiotherapy for non-small cell lung cancer
Source: J Biomed Sci. 2020 Jul 22;27:82. doi: 10.1186/s12929-020-00676-5 (PMC7374898; doi:10.1186/s12929-020-00676-5)
Supplement: Supplementary file 1 — Additional file 1: Supplementary Table 1. Ongoing or recent completed clinical trials with precision radiotherapy strategies in non-small cell lung cancer [file 12929_2020_676_MOESM1_ESM.docx]

| Supplementary Table 1. Ongoing or recent completed clinical trials with precision radiotherapy strategies in non-small cell lung cancer | | | | |
| --- | --- | --- | --- | --- |
| Trial Identifier | Modality | Phase | Study | Status |
| NCT01190527 | FDG-PET | Phase II | Effect of Midtreatment PET/CT-Adapted Radiation Therapy With Concurrent Chemotherapy in Patients With Locally Advanced Non–Small-Cell Lung Cancer. A Phase 2 Clinical Trial | Complete |
| NCT01207063 | FDG-PET | Phase II | Adaptive Radiotherapy for Stage II-III Non-small Cell Lung Cancer. | Complete |
| NCT01261598 | FDG-PET | Not Applicable | Predictive Value of FDG-TEP During Radiotherapy (RT) or Chemo-radiotherapy (CRT) in Patients With Non Small Cell Lung Cancer on the One-year Survival | Complete |
| NCT02788461 | FDG-PET | Not Applicable | Assessing the Efficacy and Safety of Selective Metabolically Adaptive Radiation Dose Escalation in Locally Advanced Non-Small Cell Lung Cancer Receiving Definitive Chemoradiotherapy (PET-BOOST) | Active  Recruiting |
| NCT02492867 | FDG-PET | Not  Applicable | A Pilot Study of Response-Driven Adaptive Radiation Therapy for Patients With Locally Advanced Non-Small Cell Lung Cancer | Active  Recruiting |
| NCT02790190 | FDG-PET | Phase III | Individualized Adaptive Radiotherapy Based on PET/CT and IMRT for Locally Advanced Non-Small Cell Lung Cancer (NSCLC) | Active |
| NCT02473133 | FDG-PET | Phase II-III | Randomized Phase II-III Study of Personalized Radiotherapy Dose Redistribution in Patients With Inoperable Stage III Non-small Cell Lung Cancer and a Persistent FDG Uptake at 42 Grays During Concomitant Radio-chemotherapy | Active  Recruiting |
| NCT02773238 | FDG-PET  SPECT | Phase II | Personalized Radiation Therapy Through Functional Lung Avoidance and Response-Adaptive Dose Escalation: Utilizing Multimodal Molecular Imaging to Improve the Therapeutic Ratio (FLARE RT) | Active  Recruiting |
| NCT01507428 | FDG-PET | Phase II | Study of Positron Emission Tomography and Computed Tomography in Guiding Radiation Therapy in Patients With Stage III Non-small Cell Lung Cancer | Active  Not recruiting |
| NCT01507428 | FDG-PET  FMISO-PET | Phase II | Randomized Phase II Trial of Individualized Adaptive Radiotherapy Using During-Treatment FDG-PET/CT and Modern Technology in Locally Advanced Non-Small Cell Lung Cancer (NSCLC) | Active  Not recruiting |
| NCT01576796 | FMISO-PET | Phase II | Phase II Study of the Efficacy and Safety of a Radiotherapy Dose Complement in the Treatment of Hypoxic Lesions Identified by F-miso PET/CT in Patients With Stage III Non-small-cell Lung Cancer (NSCLC) Not Amenable to Curative Surgical Resection Who Are Candidate for Curative Radio-chemotherapy | Active  Not recruiting |
| NCT03583723 | CT | Not Applicable | Adaptive Radiotherapy in Locally Advanced Non-Small Cell Lung Cancer (LARTIA Trial) (LARTIA) | Active  Not recruiting |
| NCT02130427 | CT / MRI | Not Applicable | A Volume, Motion, and Anatomically Adaptive Approach to Photon and Proton Beam Radiotherapy | Active  Not recruiting |
| NCT03074175 | miRNA | Not Applicable | Plasma miRNAs Predict Radiosensitivity of Different Fractionation Regimes in Palliative Radiotherapy for Advanced Non-small Cell Lung Cancer：Multicenter Controlled Study. | Not yet recruiting |
| Abbreviations: NSCLC, non-small cell lung cancer. CT, computed tomography. MRI, Magnetic Resonance Imaging. PET, positron emission tomography. SPECT, Single-photon emission computed tomography. miRNA, Micro RNA. | | | | |
